# A138 ENDOSCOPIC SUBMUCOSAL DISSECTION OUTCOMES IN T1B COLORECTAL CANCER: A SINGLE CENTER EXPERIENCE

**DOI:** 10.1093/jcag/gwad061.138

**Published:** 2024-02-14

**Authors:** F Milne, R Bechara

**Affiliations:** Queen's University, Kingston, ON, Canada; Queen's University, Kingston, ON, Canada

## Abstract

**Background:**

Endoscopic submucosal dissection (ESD) has transformed the approach to gastrointestinal neoplasms, particularly in managing T1 colorectal cancer(CRC) with superficial submucosal invasion (SMI), which is considered curative when other high-risk features are absent.

**Aims:**

The aim of this study is to describe a single center’s experience with safety, and technical and clinical outcomes for patients undergoing ESD with T1b CRC, as well as explore differences in outcomes depending on depth of SMI.

**Methods:**

This is a retrospective, single center study of patients who underwent ESD for suspected CRC at the Kingston Health Sciences Center, Ontario, Canada between October 2016 and April 2023. Patients with T1b CRC on their final pathology were included. We analyzed patient demographics, tumor characteristics, and technical outcomes. Patients were followed to determine cancer-free survival and tumor recurrence. Patients were stratified and compared by depth of SMI on their final pathology.

**Results:**

A total of 32 patients with 33 T1b CRCs were included. 19 lesions had superficial SMI, and 14 had deep SMI on final specimens. Two procedures were aborted due to suspected deep invasion or fibrosis limiting ESD. The mean procedure time was 74.5 minutes and the mean efficiency was 12.3 min/cm3. ESD had high en-bloc (90.9%) and R0 (78.7%) resection rates, and there was no significant difference regardless of depth of SMI (p=0.08 and 0.372, respectively). 24 of 33 lesions had biopsy prior to ESD, and 22 of 24 lesions with previous histology (91.7%) had upgrade in pathology on their final ESD specimen. 2 patients had adverse events, and both were managed supportively.

A total of 9 patients (27%) went on to have surgery. Those with deep SMI were more likely to have surgery (5% vs 57%, pampersand:003C0.005). The most common reason was lymphovascular invasion (44%). However, in the 7 patients who had complete ESD procedure and subsequent surgery, none had residual cancer on their surgical specimens, or malignant lymph nodes.

The follow-up period for all patients ranged from 1 to 43 months with a mean follow-up time of 14.9 months. Of those who did not have surgery, all were cancer free at a mean follow up time of 16.2 months. Within the group of patients who had surgery, all were cancer free at a mean follow up time of 11.6 months. 5 patients died during the follow up period. None were from procedural related complications or disease recurrence.

**Conclusions:**

ESD for T1b CRC is safe with high en-bloc and R0 resection rates. Patients with deep SMI were more likely to go on to have surgery, though this was frequently due to an additional high risk feature. Recurrence rates were low, but studies with longer follow up are needed.

**Funding Agencies:**

None

